# Dropout from farm-based day care for people with dementia in Norway: a follow-up study

**DOI:** 10.1186/s12877-020-01826-y

**Published:** 2020-10-27

**Authors:** T. L. Ibsen, Ø. Kirkevold, G. G. Patil, S. Eriksen

**Affiliations:** 1 Norwegian National Advisory Unit on Ageing and Health (Ageing and Health), Vestfold Hospital Trust, Tønsberg, Norway; 2 Faculty of Medicine, University of Oslo, Oslo, Norway; 3 Norwegian University of Science and Technology (NTNU) Department of Health Sciences in Gjøvik, Gjøvik, Norway; 4 Centre of Old Age Psychiatry Research, Innlandet Hospital Trust, Gjøvik, Norway; 5 Department of Public Health Science, Faculty of Landscape and Society, Norwegian University of Life Sciences, Ås, Norway; 6 Lovisenberg Diaconal University College, Oslo, Norway

**Keywords:** Dementia, Day care, Farm-based day care, Care farms

## Abstract

**Background:**

Farm-based day care services (FDCs) for people with dementia are intending to provide social relationships and meaningful activities in an agricultural landscape and offer respite for next of kin. As this requires a certain cognitive and physical functioning, it is of interest to investigate how this service contribute during the course of dementia. In this study we aim to explore the individual characteristics predicting dropout from FDC. Furthermore, we investigate whether the participants who drop out of the service continue to live at home with another day care service or if they move to a residential care facility.

**Methods:**

The study includes 92 people with dementia attending FDCs in Norway, assessed with standardized instruments at baseline between January 2017 and January 2018. They were followed for 1 year, and dropouts from FDC during this period were mapped. The association between individual characteristics and dropout was assessed using a Cox proportional hazards regression analysis.

**Results:**

Thirty-eight people stopped attending FDCs during the study. Twenty-six moved to residential care. Among the 12 who continued to live in their own homes, 9 people started in a regular day care service. Higher score on educational level and more severe dementia, as well as lower scores on social support, increased the probability of stopping FDC.

**Conclusion:**

FDCs appeared as a service that is stable over time for most participants, as more than two-third could use the care facility until the need of residential care. The transfers within care services and levels of care seemed to be characterized by continuity. More research on the growing population of educated older adults with dementia are warranted, to facilitate for their course of care needs. Finally, extended knowledge is needed to improve the collaboration between private and public networks, such as day care services, to improve the experience of social support for people with dementia.

## Background

Most people with dementia wish to live in their own home for as long as possible [1], and it is a global political goal to support this [2–4]. To achieve this, people with dementia and their next of kin need assistance to handle the challenges in everyday life that occur as the dementia progresses [5, 6]. Stephan et al. [7] emphasize the need for stability in the services offered and carefully planned transitions between levels of care. People with dementia are vulnerable to changes in daily life and threats to their independence, and factors such as stability and social support are important to reduce emotional distress [7, 8].

In Norway, approximately 80,000 people have dementia. It is estimated that about 60% of these live in their own home [9]. Day care services are a common type of service offered for home-dwelling people with dementia in Norway. In 2018, 87.7% of the Norwegian municipalities offered day care services for a total of 7909 people with dementia, between one and 5 days a week. From 2020, all Norwegian municipalities are obliged to offer day care services to the target group [9]. Day care services intend to support the maintenance of functions of daily living among the participants, provide meaningful activities and social relations with others, and offer respite for next of kin [2, 10]. Most day care services are located in a health care institution [11]. Interviews with both the participants [12] and next of kin [5] have revealed that day care services meet these intentions, and that the participants experience stability in the structure of the day by attending the service [12]. However, regular day care services have been criticized for providing too sedentary activities, and they do not adjust the intensity or type of activity to those who feel physically healthy [3, 12]. In recent years, there has been an increased interest in Norway on providing a variation in the type of day care services to meet the diversity in the population of people with dementia [2].

Farm-based day care services (FDCs) meet this request for variation by offering more diversity in activities and outdoor experiences than regular day care [13, 14]. In 2018, a survey found that 240 people with dementia attended FDC in Norway [14]. Most of the Norwegian FDCs have agricultural production and include participants in adapted farm activities such as plants and vegetables, cutting and stacking wood, raking, and taking care of animals [14, 15]. In addition, participants go for walks and take part in domestic and cooking activities [14, 16]. Norwegian FDCs are similar to regular day care when it comes to organisation, daily structure, and employees with health care education [14]. The attendants are mostly men (62%); they are younger and have a higher educational level than those in regular day care [17, 18]. Furthermore, the majority of Norwegian FDCs have people with early onset dementia or dementia in an early phase as their primary target group, and they require a certain level of physical functioning of the participant [14].

The Norwegian government’s health care strategy emphasizes that each individual shall receive care in line with the person’s care needs. This is visualized by stair steps, where the care recipient starts on the lowest step (lowest level of care) and is moved up in accordance with an increased need [19]. It is of interest to explore how FDC could contribute to this model of care provision. We have not found studies investigating predictors for dropout from neither FDC nor regular day care. However, studies on whether regular day care services postpone nursing home admission have revealed that participants often attend regular day care until they are transferred to nursing homes [20, 21]. Nursing home admissions for people with dementia are related to higher age, neuropsychiatric symptoms, functional impairment, living alone [20, 22, 23], and caregiving stress for next of kin [6, 23]. Moreover, educational level as a proxy for cognitive reserves is found to expedite nursing home admission, as highly educated persons exhibit faster cognitive deterioration in the severe stage of dementia [24]. There is no knowledge about whether FDC participants are transferred to residential care when they stop attending FDC, or if they drop out for other reasons.

In the present study, we will explore the individual characteristics predicting that people with dementia will stop attending FDC. Furthermore, we will investigate whether the participants who drop out of the service continue to live at home with another day care service or if they move to a residential care facility. This knowledge is of value to understand risk factors for stopping the service, and for planning suitable care for people with dementia.

## Methods

### Design and participants

The present study is part of a larger project studying people with dementia attending FDCs in Norway [25]. A total sample of 94 people with dementia attending FDC at 25 different farms in Norway, and their next of kin, were recruited from January 2017 to January 2018. The recruiting process is described in detail in Ibsen et al. [17], showing that of the 240 participants in Norwegian FDCs, 169 dyads met the inclusion criteria. Sixty-two dyads did not want to participate, and 13 dyads were not asked for participation. The people with dementia had attended FDC for a minimum of 3 weeks, lived in their own home, and had a next of kin who met them at least once a week. Both persons in the dyad had to be willing to participate. In the present study, we mapped those who stopped attending FDC. Of the 94 participants in the research program, one participant was excluded from the analysis due to incorrect registration of time for assessment. Another was excluded because the dropout was a result of the FDC being closed and not influenced by the characteristics of the participant. Thus, the study was conducted with 92 cases.

### Data collection

The data used for this study were collected at the recruitment (baseline) of participants to the larger project [25]. All participants were followed-up for 1 year after baseline, +/− 1 month. For those who stopped attending the service within this time frame, the date they stopped, the reason why and further major care service provided were registered. This information was given by the FDC provider by phone to the researchers in the research program.

The data collection and the standardized assessment instruments are described in detail elsewhere (Ibsen et al., 2019). For the present study we included instruments that have been reported to predict transfer to residential care or death of people with dementia [20, 22, 23]. Additionally, we included an instrument for perceived social support (The Oslo Social Support form), as it is important for psychological distress and depressive symptoms [26], somatic health [27] and the feeling of fellowship in the day care services [12, 15]. The following instruments were used in the present study:

The Clinical Dementia Rating scale (CDR) [28] assesses the cognitive and functional performance in six areas and is scored by the assessor who considers all available information. In the analysis, we used the CDR sum of boxes (CDR-SOB). The CDR-SOB scores ranges from 0 to 18, indicating questionable cognitive impairment, mild, moderate, and severe dementia [29]. CDR is validated and has good internal consistency and internal responsiveness [30].

The Neuropsychiatric Inventory (NPI) questionnaire is a 12-item instrument assessing neuropsychiatric symptoms [31]. The questionnaire is validated and tested for reliability for assessing people with dementia. The psychometric properties are found to be satisfactory [31–33]. The NPI is rated based on the next of kin’s observations over the previous month. Three NPI sub-scores were calculated according to earlier research denoted as NPI psychosis (delusions and hallucinations), affective (depression, anxiety and apathy), and hyperactivity (agitation, disinhibition, euphoria, irritability, and aberrant motor behaviour) [22].

The General Medical Health Rating (GMHR) scale [34], assesses somatic health based on how the participants’ physical health appears, and the number of medications prescribed. The score is produced in one of four categories: poor, fair, good, and excellent. GMHR was assessed by the researcher. The GMHR scale is found to be valid and reliable in studies with people with dementia [34].

The Physical Self-Maintaining Scale (PSMS) assesses functional impairment in activities of daily living. PSMS contains 5 items and the sum score ranges between 6 and 30 [35]. A higher score indicates lower functioning. The PSMS was assessed by next of kin. Reliability and validity of PSMS is found to be good for older adults [36].

The Oslo Social Support (OSS-3) form [37] assesses the participants’ self-reported experience of social support using three questions. The sum score ranges from 3 to 14, grouped into three categories: poor, moderate, and strong support [27, 37]. The OSS-3 form is found to be valid for population studies [26] and are recommended by WHO for assessing social support [38].

In addition, information about demographic data such as age, sex, marital status, and educational level was gathered by asking the participant and their next of kin.

### Statistical analysis

We conducted the statistical analysis using IBM-SPSS version 26 [39]. Missing values in the different instruments were imputed on the item level for the cases with at least 50% of the items available. Imputed values were random numbers drawn from the observed distribution in the dataset. OSS-3 was the item most imputed (9 cases). Correlations were tested using Pearson’s correlation. Demographics and characteristics were presented as frequencies, percentages, means, and standard deviations (SDs), as appropriate. A one-way ANOVA analysis with post hoc tests was conducted to compare the three groups: 1) those who stayed at FDC, 2) those who stopped but continued to live at home, and 3) those who moved to residential care. The post hoc tests showed that groups 2 and 3 differed from group 1, but not from each other. Thus, further analysis was conducted between those who stopped and those who continued FDC. Chi-square tests and independent samples t-tests were used to compare those who stopped attending FDC with those who did not.

We computed a time-variable from the time of baseline assessment to ending FDC (time from assessment), which was used as time variable in our analysis. In addition, we noted the time from starting to ending the attendance in the FDC (time from start up at the FDC) to describe total time spent in the service.

**Fig. 1:**
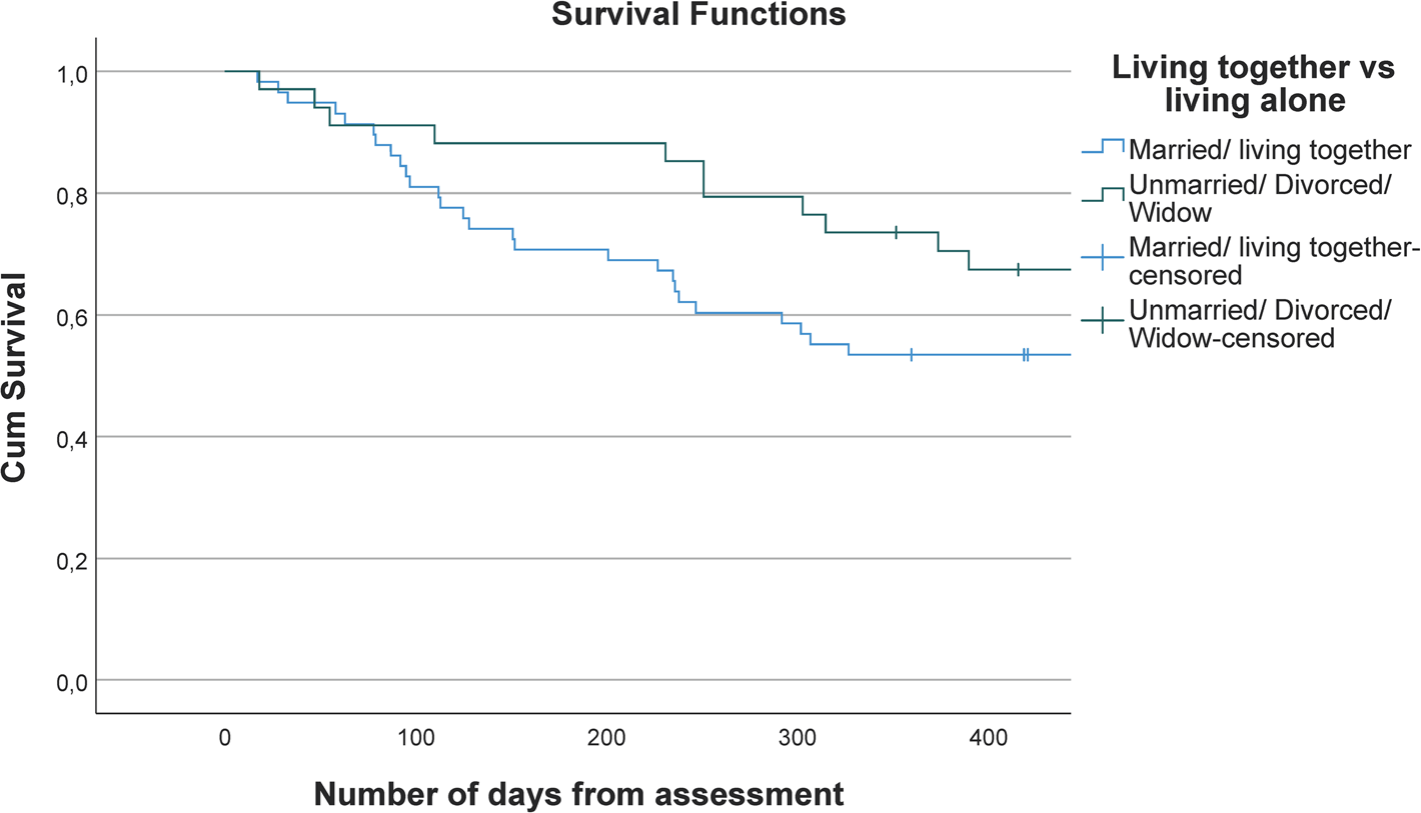
Illustration of non-crossing lines (marital status), which show that the variable is independent of time

To assess the association between the described variables and stopping attending FDC, Cox proportional hazards regression analysis was performed initially in univariate models, and then in a multivariate model. Finally, to ensure that the effects of the explanatory variables were independent of time from baseline, we estimated the Cox model for the continuous variables (age, CDR-SOB, NPI sub-scores, GMHR, PSMS, OSS-3) and saved the partial residuals. In Cox-regression analysis it is assumed that the hazard ratio should be constant across time, thus, we tested the correlation between the partial residuals and the time variable. Variables with correlations < 0.2 were judged to be independent of time and retained in the model [40], which was the case for all the variables included. For the dichotomous variables (sex, marital status, education), we plotted a survival curves with a Kaplan-Meier plot, split on the two possible scores. Non-crossing lines on the variable were assumed to be independent of time [40], which was the case for our dichotomous variables. This is illustrated by the Kaplan-Meier plot on marital status (Fig. 1).

## Results

The present study included 92 people with dementia who attended FDC. Of these, 38 people stopped attending the farm during our 1 year follow up. The baseline characteristics of the participants are described in Table 1.

**Table 1:** Baseline characteristics of the participants at farm-based day care (FDC), comparing those who stopped attending FDC and those who continued at the FDC during the one-year follow-up period

Characteristics	Stop attending FDC (*N* = 38)	Continue attending FDC (*N* = 54)	*P*-value
Sex, number (%)			
Male	25 (64.1)	32 (59.3)	0.525(chi-square)
Female	13 (35.9)	22 (40.7)	
Marital status, number (%)			
Married/cohabitant	27 (71.8)	31 (57.4)	0.182 (chi-square)
Alone (single, widowed)	11 (28.2)	23 (42.6)	
Education, number (%)			
Primary school	8 (21.1)	22 (40.7)	**0.047**(chi-square)
Higher education	30 (79.9)	32 (59.3)	
Age, mean (SD)	76.8 (7.4)	74.9 (8.8)	0.299 (t-test)
Clinical dementia rating, (CDR-SOB), mean (SD)	9.1 (3.3)	6.2 (2.4)	**< 0.001** (t-test)
Neuropsychiatric symptoms (NPI), mean (SD)	16.0 (14.8)	9.1 (10.5)	**0.014** (t-test)
- psychosis	1.1 (1.5)	0.6 (1.0)	0.061
- affective	1.9 (1.7)	1.4 (1.8)	0.167
- hyperactivity	2.9 (2.1)	1.5 (1.6)	**0.001**
General medical health (GMHR), mean (SD)	3.3 (0.7)	3.2 (0.8)	0.702 (t-test)
Physical Self-Maintaining Scale (PSMS), mean (SD)	10.4 (3.7)	8.3 (2.4)	**0.003** (t-test)
Social support (OSS-3), mean (SD)	10.4^a^ (2.0)	11.3 (1.9)	0.061 (t-test)

*N* Number of participants with complete data

^a^*n* = 27 on Social support for those who stop attending FDC

Bold values indicates statistical significance, *p*-value < 0.05

Those who stopped attending FDC had a higher educational level than those who continued at the FDC. Furthermore, at baseline, they had significantly higher scores for dementia severity (CDR-SOB), neuropsychiatric symptoms (NPI mean and NPI hyperactivity), and activities of daily living (PSMS).

Table 2 presents the results of the Cox regression analysis. In the univariate analysis, we found that higher scores in educational level, more severe dementia (CDR-SOB), neuropsychiatric symptoms (NPI psychosis and hyperactivity), activities of daily living (PSMS), and lower score in perceived social support (OSS-3), were associated with dropout from FDC. The multivariate Cox proportional hazard regression model showed that a higher score on educational level and more severe dementia (CDR-SOB), as well as lower scores on social support (OSS-3), increased the probability of stopping FDC (Table 2). Specifically, those with higher education had a 3.7 times hazard for stop attending FDC than those who had only undertaken primary school. In our material the range in dementia severity (CDR-SOB) was from 3 to 16 points, and for each step on the scale it was a 1.2 times hazard for drop out of FDC. In regard of perceived social support (OSS-3), which ranged from 4 to 14, each step on the scale showed an increase of 0.8 times (20%) reduced hazard for drop out.

**Table 2:** Cox proportional hazards regression model of individual characteristics that predict that people with dementia stop attending FDC

Characteristics	Univariate analysis HR (95% CI) *n* = 92^a^	p-value	Multivariate analysis HR (95% CI) *n* = 81	*p*-value
Sex (ref = female)	1.172 (0.609–2.255)	0.635	2.092 (0.729–6.008)	**0.170**
Marital status (ref = married)	1.732 (0.861–3.484)	0.110	1.669 (0.562–4.956)	**0.356**
Education level (ref = primary school)	2.216 **(1.015–4.838).**	**0.046**	3.748 **(1.153–12.181)**	**0.028**
Age	1.019 (0.982–1.057)	0.330	1.020 (0.959–1.085)	**0.527**
Clinical Dementia Rating (CDR-SOB), mean (SD)	1.251 **(1.143–1.369**)	**< 0.001**	1.222 **(1.002–1.491)**	**0.048**
Neuropsychiatric symptoms (NPI), mean (SD)				
- psychosis	1.228 **(1.003–1.502)**	**0.046**	0.891 (0.50–1.220)	**0.471**
- affective	1.124 (0.959–1.318)	0.150	1.007 (0.808–1.255)	**0.951**
- hyperactive	1.294 **(1.110–1.507)**	**0.001**	1.234 (0.987–1.543)	**0.065**
General medical health (GMHR), number (%)	1.110 (0.721–1.708)	0.635	1.198 (0.579–2.481)	**0.628**
Physical Self-Maintaining Scale (PSMS), mean (SD)	1.192 **(1.091–1.301)**	**< 0.001**	1.022 (0.859–1.216)	**0.808**
Social Support (OSS-3), mean (SD)	0.823 **(0.695–0.976)**	**0.025**	0.756 **(0.586–0.959)**	**0.021**

*n* Number of participants with complete data

^a^*n* = 92 for all variable except for OSS-3 (*N* = 81)

*HR* Hazard Ratio

Bold values indicates statistical significance, *p*-value < 0.05

**Fig. 2:**
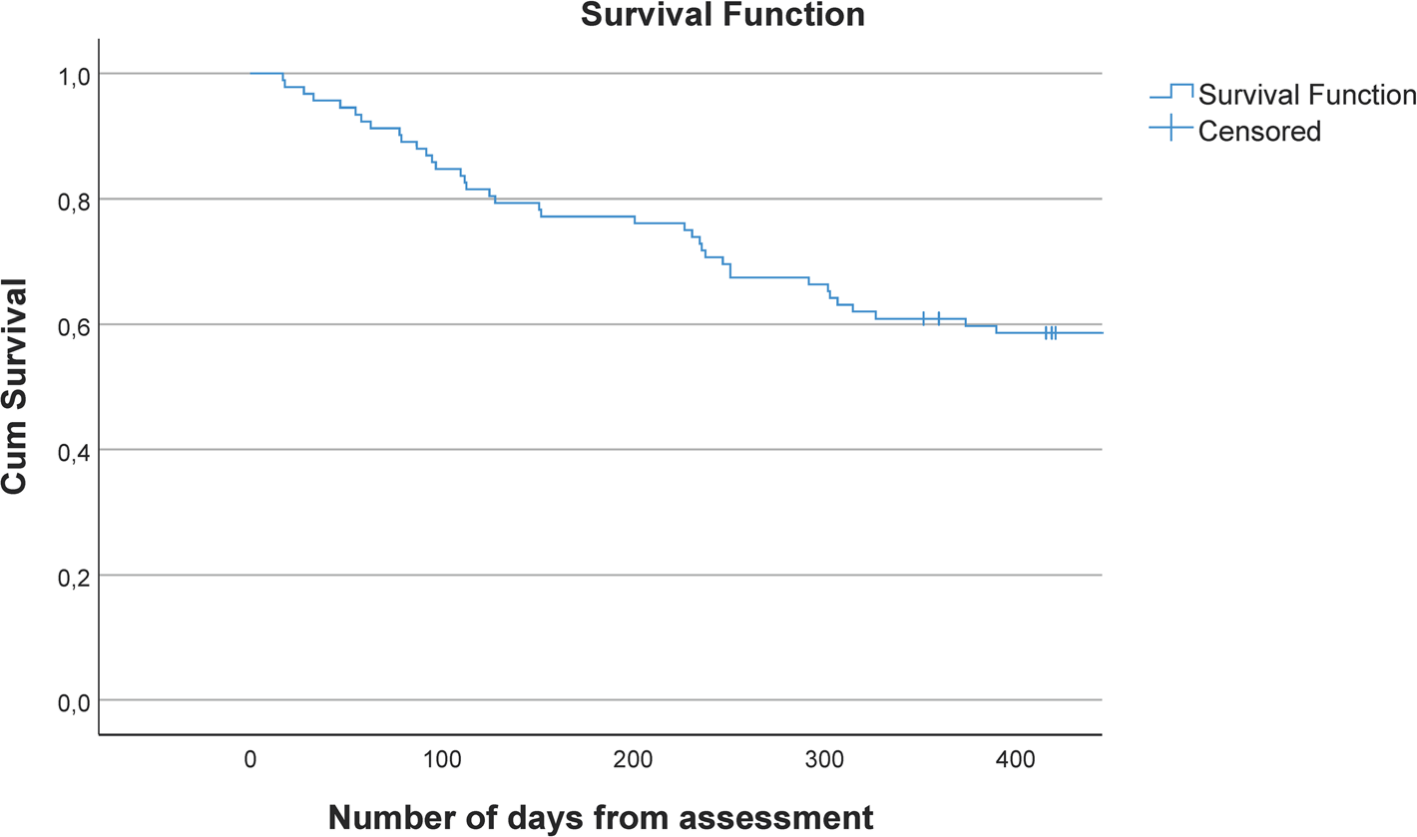
Time from baseline until dropout from farm-based day care (FDC)

Of the 38 who stopped attending FDC, 26 moved to residential care and 12 continued to live in their own homes. Of these, three stopped attending any day care and nine started at a regular day care service. Of the nine transferred to regular day care, four stopped at FDC due to cognitive and physical decline, three stopped due to physical decline only, and two people did not find FDC suitable. The mean number of days from assessment date to dropout was 170 days (range 17–390 days) (Fig. 2). For those who stopped attending FDC, the mean number of days from start of the service to dropout was 661 (range, 137–1854 days).

## Discussion

The present study aimed to identify individual characteristics predicting that people with dementia stop attending FDC. Furthermore, among those who stopped attending FDC, we wanted to investigate whether they continued to live at home with another day care service or if they moved to a residential care.

### Predictors for stopping farm-based day care

Our findings reveal that higher educational level, more severe dementia, and lower social support predict that participants stop attending FDC.

It has been claimed that people with higher education have more cognitive reserves than those with lower education, and thus compensate for the cognitive decline longer. Consequently, when people with higher education have symptoms of dementia, the symptoms seem to develop faster and the need for nursing home increases [24]. In the present study, educational level was maintained as a predictor for dropout even when adjusting for other variables. One possible explanation is that those with higher educational level than primary school more strongly request a different type or higher level of care when they feel the need. It is likely that their next of kin are also resourceful and do the same. Our study cannot answer this, but such questions and knowledge of educational influence must be taken into account when planning further care for people with dementia, as more of the growing population of older adults have a higher level of education [41].

Not surprisingly, dementia severity was a predictor for dropout, just as it is an important predictor for nursing home admission [22, 23]. Although the person may continue to live in their own home after dropping out from FDC, a transfer to regular day care may also indicate a need for a more comprehensive care. In Norway, regular day care service most often offers service more days per week than FDC [11, 14]. More days with service are often needed for continuity and extended care for persons with dementia, due to impairment in activities of daily living and neuropsychiatric symptoms. In addition, more severe dementia invokes an increased need for help and respite for next of kin [5, 6]. Our study found that activities of daily living and neuropsychiatric symptoms were not significant predictors for stopping FDC when adjusting for other variables. However, the sub-category NPI hyperactivity is close to significant. We will need a study with more participants to determine whether this is a tendency or an arbitrary finding.

The last predictor for discontinuing FDC was low social support. People with dementia report that they often experience their social network withdrawing due to the dementia diagnosis [42]. Lack of social support is found to influence psychological distress, depressive symptoms, and life satisfaction [26]. These elements are known to affect a person’s level of energy and the ability to perform activities [43]. Furthermore, there is a close relationship between people with dementia’s experience of social support and whether the next of kin experience social support in their lives [44, 45]. This is a consequence of people with dementia becoming more dependent on their next of kin during the course of dementia [42]. At the same time, it has been reported that the next of kin often experience a reduction or even absence of social support in the same period [46]. Thus, they are left with the person with dementia as their main relation and a large responsibility for the care of the same person. Mafioletti et al. (2019) found that low quality in the dyad’s relationships expedited institutionalization. Day care services are claimed to provide social support for both the person with dementia [12, 15] and to some extent for next of kin [5]. However, the findings in the present study indicate that attending day care may not be enough to prevent the feeling of lack of social support.

### Further care after drop-out from farm-based day care

Keeping in mind the main target group in FDC (people in early phase dementia with a certain functional level), it is of interest that as many as two-thirds of those who stopped attending FDC moved directly to residential care. This can be interpreted as the FDC manage to facilitate the service to the cognitive and functional level of the participant to some extent, even when the dementia severity progressed. Dutch and Norwegian studies support this finding, pointing out that the variety of activities provided at an FDC makes it easier to offer individually tailored services [14, 47]. As dementia develops and the need for care increases the change to a higher level of care is often inevitable. In this process, continuity and avoidance of fragmented services are important for people with dementia [7]. Some of those who no longer could stay at the FDC due to physical and/or cognitive decline, started in a regular day care service. Although the present study did not map how transfer between levels of care was performed, the direct transitions to other care services may witness a certain continuity as described by Stephan et al. (2018). This is in line with the Norwegian health care strategy (described with different “stairs” of care) that emphasizes continuity, though it involves some fragmentation of the services [19].

Additionally, we found that a few participants stopped at FDC because they did not find the service suitable, or did not want any type of day care.. This may indicate that they, or their next of kin, had some influence on whether they wanted to stay at the day care service or not. People with dementia, as everybody else, emphasize the importance of taking part in decisions regarding their own care and care needs. At the same time, qualitative studies have revealed that those who do not use the services offered often regret this in later stages of the condition [3]. Therefore, it is important to offer a variety of types of day care services that are attractive to different groups in the population with dementia, even if it generates greater requirements to ensure good transitions.

### Strengths and limitations

The findings in the present study must be interpreted with caution as the number of participants was low. This may have influenced the statistical analysis, and the level of significance (e.g., NPI hyperactive, which was close to significant in our material, is most often a significant predictor for nursing home admission [22, 23]. Similarly, descriptions of further care are derived from observations in a small sample. Moreover, it is likely that variables such as educational level and social support are associated with aspects other than those discussed here, which may also have influenced whether the participants stayed at an FDC service or not.

## Conclusions

The findings of the present study showed that higher educational level and dementia severity, and lower scores in social support predict dropout from FDC. FDC services seem to represent stability in everyday life for most participants, as many could stay in the day care until they needed residential care. The transfers within care services and levels of care seemed to be characterized by continuity to some extent. Knowledge about the course of care needs for higher educated people with dementia is important for facilitating services for the growing population of educated older adults, and more research must be undertaken on this group. When it comes to social support, it is necessary for actions that strengthen the entire network around the person with dementia. Support provided by the FDC is not enough to meet the increased need of the person with dementia and their next of kin, as the dementia develops. More research is needed to find the best model of collaboration between private and public networks, such as day care services, to promote stability and continuity for people with dementia.

## Data Availability

The datasets generated and analyzed during the current study are not publicly available for the sake of anonymity, due to the small number of participants. It may be available from the corresponding author on reasonable request.

## Abbreviations

FDC: Farm-based day care
CDR: The Clinical Dementia Rating scale
CDR-SOB: The Clinical Dementia Rating scale sum of boxes
NPI: The Neuropsychiatric Inventory questionnaire
GMHR: General Medical Health Rating scale
PSMS: The Physical Self-Maintaining Scale
OSS-3: The Oslo Social Support form
